# Fecal Microbiota Transplantation Controls Progression of Experimental Autoimmune Hepatitis in Mice by Modulating the TFR/TFH Immune Imbalance and Intestinal Microbiota Composition

**DOI:** 10.3389/fimmu.2021.728723

**Published:** 2021-11-29

**Authors:** Ma Liang, Zhang Liwen, Song Jianguo, Dai Juan, Ding Fei, Zhang Yin, Wu Changping, Chen Jianping

**Affiliations:** ^1^ Department of Gastroenterology, The First People’s Hospital of Changzhou, The Third Affiliated Hospital of Soochow University, Changzhou, China; ^2^ Department of Gastroenterology, The People’s Hospital of Wuqia, Xinjiang, China; ^3^ Department of Pediatrics, the Second People’s Hospital of Changzhou, Affiliate Hospital of Nanjing Medical University, Changzhou, China

**Keywords:** fecal microbiota transplantation, TFR cells, TFH cells, AIH, EAH

## Abstract

Intestinal microbiota (IM) dysbiosis contributes to the development of autoimmune hepatitis (AIH). This study aimed to investigate the potential effect of fecal microbiota transplantation (FMT) in a murine model of experimental AIH (EAH), a condition more similar to that of AIH patients. Changes in the enteric microbiome were determined in AIH patients and EAH mice. Moreover, we established an experimental model of secondary EAH mice harboring dysbiosis (ABx) to analyze the effects of therapeutic FMT administration on follicular regulatory T (TFR) and helper T (TFH) cell imbalances and IM composition *in vivo*. Alterations of the IM composition and bacterial translocation occurred in AIH patients compared to nonalcoholic fatty liver disease patients and healthy controls (HCs). Therapeutic FMT significantly attenuated liver injury and bacterial translocation and improved the imbalance between splenic TFR cells and TFH cells in ABx EAH mice. Furthermore, therapeutic FMT also partially reversed the increasing trend in serum liver enzymes (ALT and AST) of CXCR5−/−EAH mice on the 28th day. Finally, therapeutic FMT could effectively restore antibiotic-induced IM dysbiosis in EAH mice. Taken together, our findings demonstrated that FMT was capable of controlling hepatitis progression in EAH mice, and the associated mechanism might be involved in the regulation of the TFR/TFH immune imbalance and the restoration of IM composition.

## Introduction

Autoimmune hepatitis (AIH) is a chronic, progressive, and immunologically mediated inflammatory liver disease (1, 2). At present, the pathophysiology of AIH is not fully unknown. Several hypotheses have been proposed that genetic susceptibility with environment factors led to the breakdown of the hepatic immune homeostasis (3), which in collaboration induce follicular helper T (TFH) cell immune-mediated necroinflammation and tissue destruction of the liver (4, 5). Our previous study also confirmed that TFH cell has been detected in the peripheral circulation, and the excessive activation of TFH cells are associated with hypergammaglobulinemia, which accelerated the immunopathological process of AIH (6). In addition, our own previous work demonstrated that follicular regulatory T (TFR) cell could inhibit the secretion of autoantibody by indirectly inhibiting the activation of TFH cells *via* the coreceptor CTLA-4. Further research found that dysregulation between TFR and TFH cells might cause excessive production of autoantibodies and destruction of the immune homeostasis, leading to the immunopathological process in AIH, suggesting that reversing the disorder between TFR and TFH cell will provide new perspectives for the treatment of AIH (6). However, recent research found that the defensive mechanism maintaining the hepatic immune homeostasis mainly depended on the intestinal microbiota (IM) and their metabolic byproducts constituting a reservoir of foreign antigens (7–10). This intimate relationship existing between the gut and liver was called “gut–liver axis”, which played a critical role in the pathogenesis initiation and progression of many types of liver diseases (11–13). There was evidence that alterations in the composition of the intestinal microbiome (dysbiosis) were found in AIH, including a reduction in microbial diversity and a reduced number of anaerobes such as *Bifidobacterium* and *Lactobacillus* (7). Furthermore, germ-free mice are resistant to concanavalin A (ConA)-induced liver injury (14), further confirming the necessity of IM in the initiation of AIH. Therefore, remodeling the homeostasis between the host and intestinal microorganisms and reversing the disorder between TFR and TFH cell will provide new perspectives for the treatment of AIH.

Corticosteroids and immunosuppressants are the main therapeutic methods for alleviating symptoms and prolonging life in AIH patients (15). Although the efficacy of these therapy are satisfactory in most patients, 10%–20% of cases with AIH still progress to end-stage liver disease and require liver transplantation (16). Furthermore, side effects of the drug—such as moon face, central obesity, and osteoporosis—might severely interrupt patients’ quality of life (17). Therefore, the development of new safe and effective therapies is an urgent need for patients with AIH.

Fecal microbiota transplantation (FMT) is a treatment method in which the functional bacteria from a healthy donor feces is transferred into another patient’s gastrointestinal tract through different routes of administration, so as to reconstitute of a healthy microbial ecosystem in the gut (18, 19). The initial purpose of FMT is used to treat intestinal-associated diseases, such as refractory and recurrent *Clostridium difficile* infections (CDI) (20). Lately, therapeutic application of FMT can improve insulin sensitivity in patients suffering from metabolic syndrome (21). Furthermore, recent studies have suggested that FMT can increase intestinal microbial diversity and modulate intestinal immune response towards control of intestinal inflammation in patients with inflammatory bowel disease, such as ulcerative colitis (UC) (22). Specifically, a recent study showed supplement sodium butyrate, a metabolite of intestinal flora, attenuated liver injury and prevented migration of intestinal pathobionts into the liver in a mouse model of AIH (10). Moreover, FMT can increase the concentration of intestinal butyrate (23). Taken together, these studies indicate that FMT may be a promising method for managing AIH. However, whether FMT can control hepatitis progression of AIH or not requires further research.

Here, the present ongoing study aims to evaluate whether FMT regulate TFR/TFH cell disorders and affect colonization of intestinal flora, so as to clarify the beneficial mechanism of action of FMT in AIH.

## Materials and Methods

### Participants

A total of 32 newly diagnosed patients with AIH were enrolled into this study at the Department of Inpatient Clinic of the Department of Gastroenterology, the First People’s Hospital of Changzhou. According to the international criteria for the definitive diagnosis of AIH type I (3), all patients were diagnosed in an active disease state, as defined by an alanine aminotransferase (ALT) value or aspartate aminotransferase (AST) value >50 U/ml. Individuals were excluded if she/he had a history of another autoimmune disease, recent infection, or received immune suppressive or glucocorticoid therapies within the past 6 months. For comparison, another 20 age-, gender-, and ethnicity-matched nonalcoholic fatty liver disease (NAFLD) patients without other disease and 20 age-, gender-, and ethnicity-matched healthy controls (HCs) without any disease were recruited from the Department of Medical Examination Center of the First People’s Hospital of Changzhou during the same period. An informed consent was written from individual participants, and the experimental protocol was approved by the Ethical Committee of the First People’s Hospital of Changzhou.

### Clinical Index Assay

The clinical data of each subject were collected from the hospital records. These data included age, sex, and laboratory tests. Individual subjects were subjected to routine laboratory tests for full blood cell counts, and the concentrations of total bilirubin (TBIL), albumin (ALB), IgG, IgM, IgA, and antinuclear and smooth muscle antibody (ANA/SMA) by a biochemistry automatic analyzer (Roche Diagnostics, Branchburg, USA) and scattered turbidimetry on a Siemens special protein analysis instrument (Siemens Healthcare Diagnostics Products, GmbH, Germany).

### Animals

Specific pathogen-free (SPF)-class female C57BL/6 mice (6–8 weeks of age, 18–22 g) and CXCR5-deficient (CXCR5−/−) mice (6 weeks of age, 16–20 g) were obtained from Nanjing Experimental Animal Center (Jiangsu, China). All animal experiments had been approved by the Laboratory Animal Ethics Committee of the First People’s Hospital of Changzhou, and the Guide for the Care and Use of Laboratory Animals were observed.

### Induction of Experimental Autoimmune Hepatitis (EAH)

According to the previously mature method of establishing autoimmune hepatitis (EAH) mouse model (5, 6), owning to AIH occurring more frequently in women, SPF class female C57BL/6 mice were selected and induced into EAH model mice, a condition more similar to that of AIH patients. Liver antigens were always prepared freshly as described previously from C57BL/6 female mice after perfusion of livers with phosphate-buffered saline (PBS). Livers were homogenized on ice, and the nuclei and remaining intact cells were centrifuged at 150*g* for 10 min. Subsequently, the supernatants were centrifuged for 1 h at 100,000*g*, and the remaining supernatants were used for immunization (called S100). Induction of experimental EAH was achieved by intraperitoneal injection of the mice with freshly prepared S-100 antigen at a dose of 0.5–2 mg/ml in 0.5 ml PBS that had been emulsified in an equal volume of complete Freund’s adjuvant (CFA) on day 0. A booster dose was given on day 7 as well (5, 6, 20). Disease severity was assessed histologically on day 28 when the peak of disease activity was observed. Disease severity was graded on a scale of 0–3 by a researcher who was blinded to the sample identity, as follows: grade 0, none; grade 1, mild–scattered foci of lobular-infiltrating lymphocytes; grade 2, moderate–numerous foci of lobular-infiltrating lymphocytes; and grade 3, severe–extensive pan-lobular-infiltrating lymphocytes.

### Mouse Model of Intestinal Dysbiosis Induced by Ceftriaxone Sodium

In order to counteract physiological colonization resistance, secondary mice harboring IM dysbiosis were generated as described previously (24). In brief, secondary EAH mice harboring dysbiosis (ABx) were administered 0.2 ml of ceftriaxone sodium (400 mg/ml) orally twice a day at an interval of 6 h for 7 days.

### Fecal Microbiota Transplantation

In brief, 50 g of fresh murine fecal samples was collected from 10 age- and sex-matched SPF-class C57BL/6 mice, pooled, dissolved in 250 ml sterile PBS, and the supernatant used as murine donor suspension under anaerobic conditions. Anesthetized EAH mice were treated with 0.3 ml of murine donor suspension *via* anus. Mock control mice were treated with 0.3 ml of PBS *via* anus.

### Histological Evaluation

Liver tissues from sacrificed animals were fixed with 4% (v/v) PFA, dehydrated through a graded series of sucrose, frozen in an optimal cutting temperature (OCT) compound (Tissue TCK, Miles Elkhart, IN, USA), and stored at −80°C. Five-micrometer cryostat sections were stained with hematoxylin and eosin (HE) to reveal and estimate the degree of inflammatory cell infiltration. Liver histology of EAH mice was scored using light microscopy and a modified Scheuer scoring scale, assigning scores for lobular inflammation (0, none; 1, mild-scattered foci of lobular-infiltrating lymphocytes; 2, moderate–numerous foci of lobular-infiltrating lymphocytes; and 3, severe–extensive pan-lobular-infiltrating lymphocytes).

### Flow Cytometric Analysis

Spleen mononuclear cells (SMNCs) were isolated from EAH mice by density-gradient centrifugation using Ficoll–Paque Plus (Amersham Biosciences, Little Chalfont, UK). To detect TFR/TFH cell subsets, SMNCs (1 × 10^6^/tube) were stained with BV510-anti-CD4 (0.2 mg/ml), PerCP-Cy5.5-anti-CXCR5 (0.1 mg/ml), and FITC–anti-GITR (0.1 mg/ml) in the dark at 4°C for 30 min. After being washed with PBS, the frequency of CD4+CXCR5+GITR+TFR and CD4+CXCR5+GITR-TFH in EAH mice were determined by flow cytometry analysis (LSR II Instrument BD Biosciences). The cells were gated on living lymphocytes and then gated on CD4+CXCR5+ T cells, and at least 20,000 events were analyzed by FlowJo software (v5.7.2).

### Enzyme-Linked Immunosorbent Assay

The concentrations of serum lipopolysaccharide (LPS) in AIH patients and the concentrations of serum endotoxin (ET) and diamine oxidase (DAO) in EAH mice were determined by ELISA. The levels of cytokines in the supernatant of normalized hepatic tissue weight (mice) were measured by ELISA using commercially available interleukin-10 (IL-10), transforming growth factor beta 1 (TGF-β1), and IL-22 ELISA kits (Boster, Wuhan, China) according to the manufacturer’s instructions.

### Real-Time PCR

Total RNA was acquired from the hepatic tissue at each time point from EAH mice. The expressions of transcriptional repressor Foxp3 and IL-21 were then measured through real-time quantitative RT-PCR. Total RNA was extracted from the hepatic tissue using the acid guanidinium thiocyanate-phenol-chloroform method. Total RNA of 2 μg was reverse transcribed with hexamer and M-MuLV reverse transcriptase (New England Biolabs, Ipswich, MA) according to the manufacturer’s instructions (R&D Systems). cDNA corresponding to 25 ng of total RNA was subjected to quantitative PCR (qPCR) using primer sets and TaqMan probes corresponding to murine Foxp3 and IL-21 with qPCR Mastermix. All estimated messenger RNA (mRNA) values were normalized to glyceraldehyde 3-phosphate dehydrogenase (GAPDH) mRNA levels. The sequences of the primers and TaqMan probes were as follows:

*FOXP3, forward: 5′-ATGGAGTCTGCAAGTGGCCT-′3; reverse: 5′-TCTGCTTGGCAGTGCTTGAG-′3*

*Probe sequences, 5′-ACTCTTCTGGTCCTCGAAGACCTTCTCA-′3*

*IL-21, forward: 5′-AAGATTCCTGAGGATCCGAGAAG-′3; reverse: 5′-GCATTCGTGAGCGTCTATAGTGTC-′3*

*Probe sequences, 5′-TTCCCGAGGACTGAGGAGACGCC-′3*

*GAPDH, forward: 5′-GCTCTGGCTCCTAGCACCAT-′3; reverse: 5′-GATCCACACAGAGTACTTGCGC-′3*

*Probe sequences, 5′-AGATCAAGATCATTGCTCC-′3*

Cycling conditions were 95°C for15 s, 95°C for 15 s, and 95°C for 15 s.

### Real-Time PCR of Intestinal Microbiome

Total DNA in fecal pellets of EAH mice was isolated using the QIAamp fast DNA stool mini kit (Qiagen) following the manufacturer’s protocol (R&D Systems). Moreover, the target bacteria 16S rRNA were amplified by using specific primers and a thermocycler PCR system (GeneAmp 9700, ABI, USA).

Quantitative real-time polymerase chain reaction (qRT-PCR) primers were used for amplification:

*Bifidobacterium, forward: 5′-TCTGGCTCAGGATGAACGC-′3; reverse: 5′-CACCGTTACACCGGGAATTC-′3*

*Lactobacillus, forward: 5′-TGGAAACAGRTGCTAATACCG-′3; reverse: 5′-GTCCATTGTGGAAGATTCCC-′3*

*Clostridium leptum, forward: 5′-GCACAAGCAGTGGAGT-′3; reverse: 5′-CTTCCTCCGTTTTGTCAA-′3*

*Escherichia coli, forward: 5′-CGGACTTTCTGCGTGCTAAGA-′3; reverse: 5′-CAATTGGATTTTTGACTTCTG-′3*

Cycling conditions were 95°C for 15 s; 60°C for 1 h, 60°C for 30 s, and 95°C for 40 s.

### Statistical Analysis

The difference between two independent groups was analyzed by the Kruskal–Wallis nonparametric test or Fisher’s exact test using the SPSS 18.0 software (SPSS, Inc., Chicago, IL, USA). A two-sided p < 0.05 was considered statistically significant.

## Results

### Intestinal Microbiota Dysbiosis in AIH Patients

To assess whether or not the intestinal flora has changed, 32 AIH patients, 20 NAFLD patients, and 20 healthy subjects were recruited. As summarized in **Table 1**, there were no significant differences in the distribution of age and gender among the different groups of patients. As expected, the levels of serum liver enzymes (ALT, AST, γ-GT, and ALP) and serum immunoglobulin (IgG, IgM, and IgA) were significantly higher in AIH patients than those in NAFLD and HC patients. Moreover, 23 of 32 patients had cirrhosis. Furthermore, the majority of patients with new onset AIH were seropositive for anti-ANAs and anti-SMA antibodies. These results indicate that AIH patient displayed active disease and hypergammaglobulinemia.

**Table 1:** The demographic and clinical characteristics of subjects.

Parameters	AIH	NAFLD	HC
No.	32	20	20
Age (years)	48 (37–76)	45 (35–66)	51 (41–74)
Gender/female/male	24/8	13/7	14/6
ALT (U/L)	125.9 ± 108.3*	57 ± 7.3^#^	27.2 ± 8.2
AST (U/L)	101.1 ± 53.7*	62 ± 6.8^#^	22.7 ± 5.7
γ-GT (U/L)	89.1 ± 30.3*	29.3 ± 6.5	25.1 ± 7.4
ALP (U/L)	133.4 ± 37.1*	90.5 ± 16.3	89.5 ± 23.6
Bilirubin (μmol/L)	12.5 ± 8.1*	9.8 ± 7.6	10.8 ± 6.8
Albumin (g/L)	23.8 ± 5.7	20.3 ± 5.5	25.3 ± 4.8
PT-INR	1.0 ± 0.9	1.0 ± 0.5	1.1 ± 0.6
Cirrhosis	25/32 (78.1%)*	–	–
Anti-ANA (+)	23/32 (71.8%)*	0/20 (0%)	0/20 (0%)
Anti-ANA titer	1:640 (1:80–1:10,000)	–	–
Anti-SMA (+)	2/32 (6.25%)	0/20 (0%)	0/20 (0%)
Anti-SMA titer	1:1,000 (1:160–1:3,200)	–	–
IgG (g/L)	15.9 ± 3.7*	5.8 ± 3.4	7.8 ± 2.3
IgM (g/L)	6.9 ± 1.9*	2.08 ± 1.03	2.64 ± 0.87
IgA (g/L)	4.07 ± 2.3*	1.2 ± 0.9	1.6 ± 1.1
WBC (*10^9^/L)	7.93 (5.6–11.2)*	4.55 (4.3–8.8)	5.08 (3.9–9.2)

Data shown are real case number or mean ± SD. Normal values: alanine aminotransferase (ALT), 5-40 U/L; aspartate transaminase (AST), 5–40 U/L; gamma glutamyl transferase (γ-GT), 10–60 μ/L; alkaline phosphatase (ALP), 45–125 μ/L; bilirubin, 3.4–20.5 μmol/L; albumin, 35–53 g/L; antinuclear antibody (ANA), <1:80; antimitochondrial antibodies (SMA), <1:80; IgG, 7–16 g/L; IgM, 0.7–4.6 g/L; IgA, 0.4–2.3 g/L.

HC, healthy control; AIH, autoimmune hepatitis.

*p < 0.05 vs. HC/NAFLD.

^#^p < 0.05 vs. HC.

**Figure 1:**
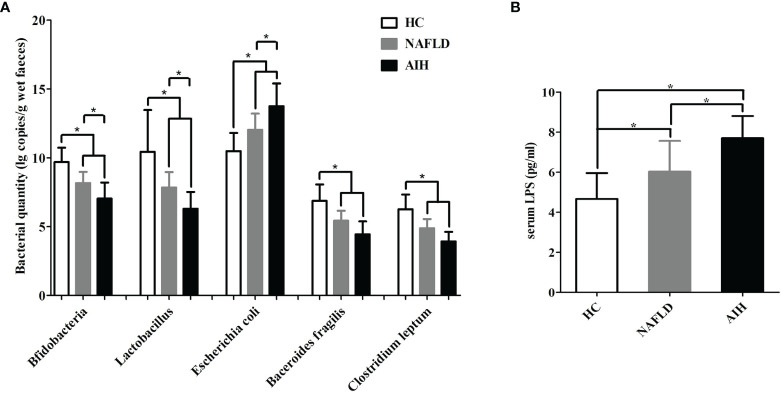
Changes in the enteric microbiome and serum LPS levels of AIH patients compared to NAFLD patients and controls. **(A)** The levels of fecal *Bifidobacteria*, *Lactobacillus*, *Bacteroides*, *Escherichia coli*, and *Clostridium leptum* l g copies/g wet feces (copies per gram of wet feces); **(B)** the levels of serum LPS. The data are presented as the mean ± SD. *p < 0.05.

Next, the major enteric bacteria in the fecal samples were quantitatively analyzed using the real-time PCR method. The quantity of *Bifidobacteria*, *Lactobacillus*, *Bacteroides*, and *C. leptum* in AIH patients were significantly lower than those in the NAFLD patients and HCs, while the quantity of *Escherichia coli* in the AIH patients were significantly higher than those in the NAFLD patients and HCs (**Figure 1A**). We further assessed the presence of bacterial translocation and found that the levels of serum LPS in AIH patients were significantly higher than those in the NAFLD patients and HCs (**Figure 1B**). These results indicate that IM dysbiosis occurred in AIH and NAFLD patients but not in healthy control group.

### Impaired Liver Function and Imbalance of TFR/TFH Cell Were More Serious in EAH Mice Harboring IM Dysbiosis

**Figure 2:**
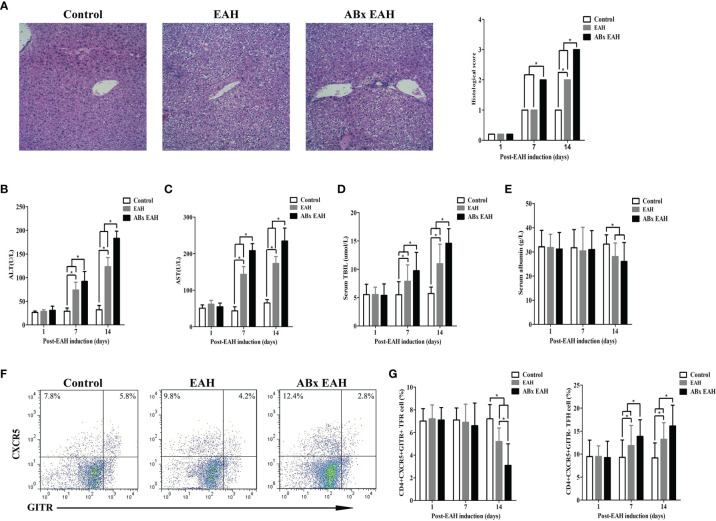
Flow cytometry of TFR/TFH cells in SMNC. Fifteen mice (five from the EAH group, five from the ABxEAH group, and five from the control group) were killed at each time point (1, 7, and 14 days). **(A)** Representative histological picture of liver lesions in animals after standard induction of ABx-EAH, EAH, and controls on the 14th day (magnification, 200×); **(B–E)** the levels of serum ALT, AST, TBIL, and ANA in mice after standard induction of ABx EAH and EAH mice with S100 and control mice with PBS; **(F)** flow cytometry analysis of TFR and TFH cells from the spleen on the 14th day; **(G)** percentages of TFR and TFH cells at each time point from ABx EAH, EAH, and control mice were analyzed by FACS. The horizontal lines indicate the mean values of the different groups. *p < 0.05.

Next, we employed S100/FCA-induced EAH model treated with broad-spectrum antibiotics (ABx) to investigate the effects of IM dysbiosis on liver functions and TFR/TFH balance. Successful alterations of the IM composition was confirmed as described previously (24). Compared with the control group and the EAH group, ABx EAH mice had obvious liver injury evidenced by liver edema with a rising liver index and elevated serum levels of ALT, AST, and TBIL and decreased serum levels of albumin (**Figures 2A**–**E**). Splenocytes were collected from mice at each time point, and flow cytometry was performed to analyze the percentages of CD4+CXCR5+GITR+TFR and CD4+CXCR5+GITR-TFH cells (**Figure 2F**). We observed that CD4+CXCR5+GITR+TFR cells in the ABx EAH group significantly decreased on the days of post-EAH induction. On the other hand, CD4+CXCR5+GITR-TFH cells in the ABx EAH gradually increased from 7th to 14th days of post-EAH induction compared to the control group and the EAH group (**Figure 2G**). These results indicated that treatment with broad-spectrum antibiotics might not only induce IM dysbiosis but also aggravate liver inflammation and TFR/TFH cell disorder in EAH mice.

### Therapeutic FMT Attenuated Liver Injury, Hypergammaglobulinemia, and Bacterial Translocation in EAH Mice Harboring IM Dysbiosis

**Figure 3:**
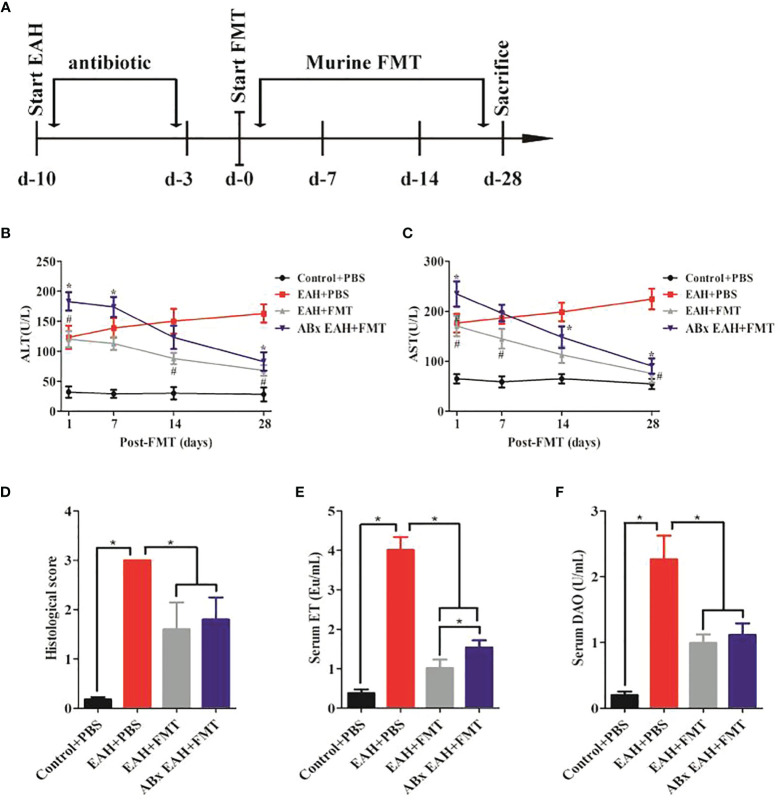
FMT attenuated liver injury and bacterial translocation. **(A)** Experimental setup. Secondary EAH mice harboring dysbiosis (ABx) mice were generated by broad-spectrum antibiotic treatment for 7 days. Three days before FMT, the antibiotic cocktail was replaced by sterile tap water to guarantee antibiotic washout. ABx EAH mice were then subjected to FMT on 28 consecutive days; **(B, C)** serum ALT/AST levels; **(D)** histological score of liver lesions; **(E)** serum ET levels; **(F)** serum DAO levels. The horizontal lines indicate the mean values of the different groups. *p < 0.05.

To evaluate if FMT might exert beneficial effects in a chronic autoimmune-related liver inflammation similar to that observed in human AIH patients, ABx EAHs were subjected to murine fecal microbiota transplantation (mFMT) for 28 consecutive days starting at day 10 antibiotic treatment. Three days before mFMT transplantation, the antibiotic cocktail was replaced by autoclaved tap water (**Figure 3A**). During the course of the treatment, the elevated serum liver enzymes (ALT and AST) of FMT-treated ABx EAH and EAH group significantly decreased on the 7th, 14th, and 28th day compared with those in the control group (**Figures 3B, C**). The histological score was also similar among FMT-treated and untreated group (**Figure 3D**). In addition, the elevated serum IgG of FMT-treated ABx EAH and EAH group significantly decreased on the 28th day compared with those in the control group (*p < 0.05). Next, we further evaluated the effect of FMT on bacterial translocation and found that the serum ET and DAO of FMT-treated ABx EAH and EAH group significantly decreased on the 28th day compared with those in the control group (**Figures 3E, F**). Thus, our results showed that therapeutic FMT might attenuate liver injury and bacterial translocation in settings that more closely resemble human AIH pathology.

### Therapeutic FMT Regulated TFR/TFH Cell Imbalances in EAH Mice Harboring IM Dysbiosis

**Figure 4:**
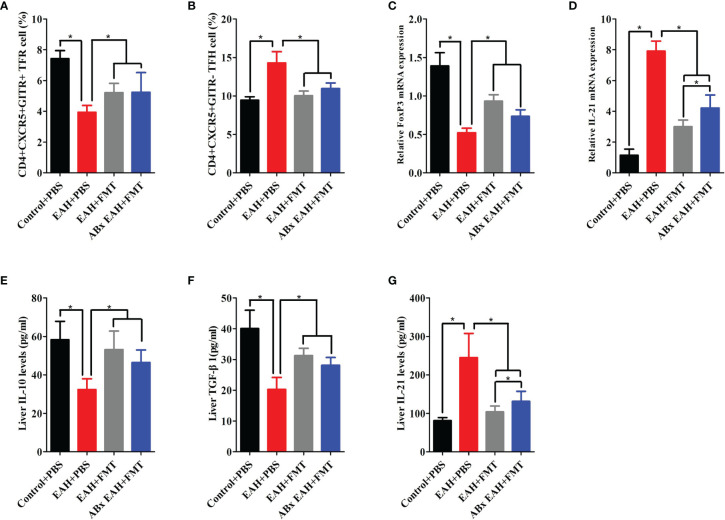
FMT-regulated TFR/TFH cell imbalances. Fifteen mice (five from the EAH group, five from the ABxEAH group, and five from the control group) were killed at each time point (1, 7, 14, and 28 days) after therapeutic FMT. **(A, B)** Percentages of TFR and TFH cells in SMNCs on the 28th day were analyzed. **(C, D)** The mRNA levels of FoxP3 and IL-21 on the 28th day were analyzed. **(E–G)** Liver IL-10, TGF-β, and IL-21 levels on the 28th day were analyzed. The horizontal lines indicate the mean values of the different groups. *p < 0.05.

To evaluate the effect of FMT on the expression of TFR/TFH cell, we further examined the impact of therapeutic FMT administration on the frequency of CD4+CXCR5+GITR+TFR and CD4+CXCR5+GITR-TFH cells in the spleen tissue of ABx EAH and EAH group. We observed that CD4+CXCR5+GITR+TFR cells and TFR/TFH ratio in the FMT-treated ABx EAH and EAH group significantly increased, while TFH cells were significantly decreased on the 28th day compared with those in the control group (**Figures 4A, B**). Furthermore, our data also showed that the liver levels of IL-10, TGF-β, and FoxP3-mRNA on the 28th day of FMT administration were statistically higher in the ABx EAH and EAH group than in the control group. Conversely, the liver levels of IL-21 and IL-21 mRNA obviously decreased on the 28th day of FMT administration compared with those in the control group (**Figures 4C**–**G**). Taken together, these data indicated that therapeutic FMT might regulate TFR/TFH cell imbalances in settings that more closely resemble human AIH pathology.

### Therapeutic FMT Attenuated Liver Injury in CXCR5−/−EAH Mice

**Figure 5:**
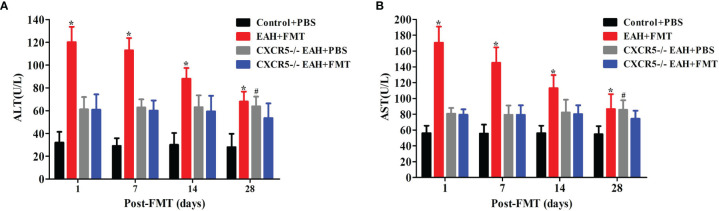
FMT attenuated liver injury *in CXCR5−/−EAH mice*. **(A, B)** Serum ALT/AST levels progressively downregulated from 1 to 28 days in EAH and CXCR5−/−EAH mice after therapeutic FMT. The horizontal lines indicate the mean values of the different groups. *p < 0.05 vs. control+PBS/CXCR5−/−EAH+PBS/CXCR5−/−EAH+FMT; ^#^p < 0.05 vs. control+PBS/CXCR5−/−EAH+FMT.

To explore whether the role of FMT in controlling hepatitis progression was achieved by regulating TFR/TFH cell balance, we employed CXCR5-deficient mice, which failed to develop discrete primary follicles. We observed that the levels of serum liver enzymes (ALT and AST) were significantly higher in PBS-treated CXCR5−/−EAH group than those in the PBS-treated control group on the 28th day. Furthermore, our data also showed that the levels of serum liver enzymes (ALT and AST) did not significantly decrease until the 28th day in the FMT-treated CXCR5−/−EAH group but decreased significantly on the 7th day in the FMT-treated EAH group (**Figures 5A, B**). These data indicated that the FMT might control hepatitis progression by regulating TFR and TFH cell imbalances, but it is not entirely dependent on this pathway.

### Therapeutic FMT Restored Antibiotic-Induced IM Dysbiosis in EAH Mice

**Figure 6:**
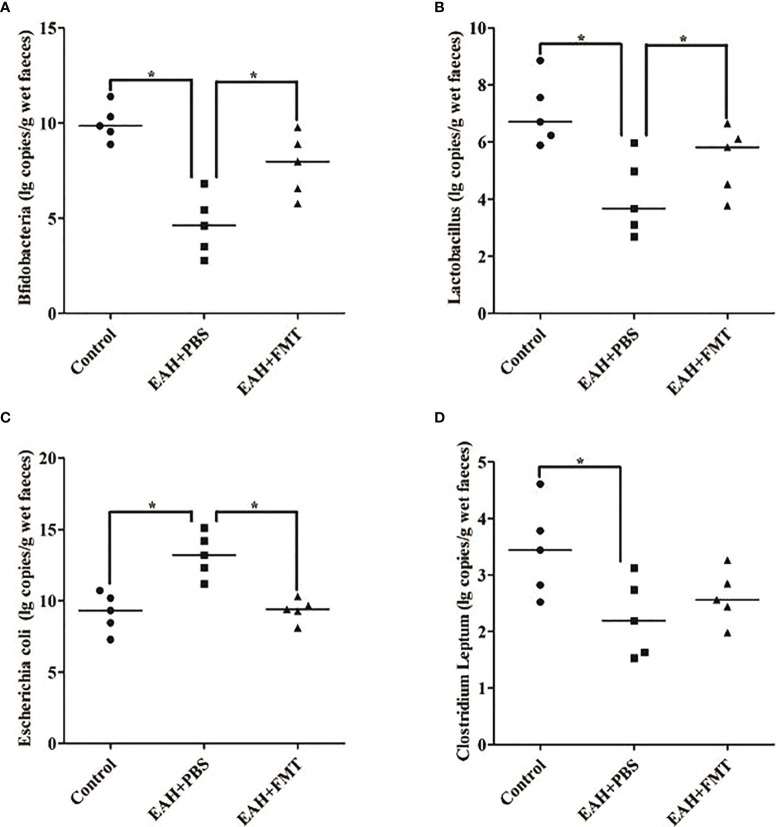
FMT restored antibiotic-induced IM dysbiosis. Fecal samples on the 28th day of untreated and FMT-treated EAH mice are analyzed by the real-time PCR method. **(A)** Amounts of fecal *Bifidobacteria*; **(B)** amounts of fecal *Lactobacillus*; **(C)** amounts of fecal *Escherichia coli*; **(D)** amounts of fecal *Clostridium leptum*. The horizontal lines indicate the mean values of the different groups. *p < 0.05.

To assess whether the reduction in liver damage in FMT-treated mice was associated with changes in the IM composition, fecal samples of untreated and FMT-treated mice were analyzed by the real-time PCR method. Compared with the control group, the quantitative amounts of *Bifidobacterium* and *Lactobacillus* were significantly increased, and *Escherichia coli* was significantly reduced on the 28th day in the FMT-treated group (**Figures 6A**–**C**). Furthermore, the quantitative amounts of *C. leptum* of the FMT-treated group did not differ from that of EAH group (**Figure 6D**). Taken together, these data indicated that the beneficial effects of FMT might originate from the functional reshuffling of the entire IM composition alterations towards anti-inflammatory activities.

## Discussion

IM dysbiosis has been associated with the pathogenesis of many autoimmune diseases, such as and diabetes mellitus type 1 (T1DM), systemic lupus erythematosus (SLE), and inflammatory bowel disease (IBD) (25, 26). Besides, various studies have also provided evidence that the liver–gut axis play an important role in the pathogenesis of chronic hepatitis, such as NAFLD and primary biliary cirrhosis (PBC) (11–13, 27). In this study, our findings revealed that the IM was disordered in AIH patients undergoing an active state. These patients had fewer *Bifidobacteria*, *Lactobacillus*, and *Bacteroides* in their intestines, suggesting that these IMs might have a preventive role in the pathogenesis of AIH. These findings were consistent with some of the results of previous studies (7), but part of the difference were that these patients had fewer *Escherichia coli* in their intestines, indicating that *Escherichia coli* might have a negative effect in the pathogenesis of AIH. Conflicting results might be due to the uncontrolled effects of factors, such as environment and eating habits. Moreover, these inconsistencies are partly due to the methodological issues that include variations in sample size and use of different techniques for the determination of the IM composition. In addition, recent studies have reported that *C. leptum* were a risk factor for the development of various autoimmune diseases, such as pulmonary immunosuppression (28) and inflammatory bowel disease (29). However, the role of *C. leptum* in AIH is still unclear. Hence, we further found lower levels of *C. leptum* in the intestines of AIH patients, indicating that their absence might be related to the pathogenesis of AIH. More importantly, consistent with previous studies (7), our data showed a high expression level of serum LPS, which are a hallmark of bacterial translocation (30), suggesting that bacteria-derived LPS translocated into the bloodstream may contribute to the progression of liver damage and fibrosis (31). Taken together, these findings support the concept that AIH might be associated with IM dysbiosis and translocation of gut-derived microbial products into the systemic circulation.

Our previous study that established an experimental EAH model in mice with S100/FCA revealed obvious liver injury evidenced by liver edema with a rising liver index and elevated serum levels of ALT, AST, and TBIL similar to that observed in human AIH (5, 6). Furthermore, our study showed that TFR and TFH cells, which were important for the maintenance of immune tolerance (32), were dysregulated in EAH mice, which was consistent with our previous studies that imbalance of TFR-to-TFH ratios promoted the pathogenesis of EAH (6). More notably, IM dysbiosis by broad-spectrum antibiotic treatment resulted in more severe liver injury and TFR/TFH imbalance in EAH mice, which further confirmed previous findings that IM disorders might play an important role in the pathogenesis of AIH (7). Therefore, the reconstruction of IM balance through administration of FMT may be a potential treatment for AIH.

In this study, we found that the onset of AIH may be also related to IM alterations. In addition, the “gut–liver axis” was widely described in various chronic liver diseases, including AIH (10). Therefore, these studies supported this concept that FMT might be a promising treatment for AIH. Here, we found that histological score of liver lesions were altered in EAH mice, and serum levels of AST and ALT in EAH mice with and without antibiotics were restored upon therapeutic FMT. Moreover, the serum ET and DAO, which are hallmarks of bacterial translocation (30, 33), were also similarly improved in EAH mice upon therapeutic FMT. Taken together, our results suggested a potential role of FMT in the amelioration of hepatic inflammatory indexes and protection of gut barrier integrity in the development of AIH.

Given that TFR/TFH cell disorder might promote the progress of AIH (6), we found that ABx EAH mice treated with broad-spectrum antibiotics exacerbated the TFR/TFH imbalance. This possible underlying mechanism of microbiota change to TFR/TFH balance is attributed to the fact that elevated LPS, a metabolite of intestinal flora, activates Toll-like receptor 4 (TLR4)/MyD88 signaling pathway, which leads to the inhibition of TFR cells and the activation of TFH cells (25). We further set out to evaluate the effect of FMT on TFR/TFH cell balance in EAH mice. Our data demonstrated dynamically elevated TFR cells and decreased TFH cells in EAH mice with and without antibiotics, indicating that the restore of IM dysbiosis induced by FMT positively promoted immune tolerance through induction of TFR cell and suppression of TFH cell. Subsequently, we employed CXCR5-deficient mice to assess whether the role of FMT in controlling hepatitis progression was achieved by regulating TFR/TFH cell balance and found that FMT was able to improve liver inflammation in CXCR5-deficient EAH mice but later than EAH mice. Collectively, our findings indicated that FMT might control hepatitis progression by regulating TFR/TFH cell balance, but it was not entirely dependent on this pathway.

Next, we found changes in the levels of *Bifidobacterium*, *Lactobacillus*, *Escherichia coli*, and *Clostridium leptum* in EAH mice, which more closely resembles the clinical features observed in AIH patients (5, 6). These findings provide strong evidence supporting the correlation between IM dysbiosis and AIH (7). However, their dysbiosis in EAH mice were partially restored upon therapeutic FMT, indicating that FMT might be beneficial to reconstruct the composition of the intestinal flora in EAH mice.

Taken together, the presented data described the IM dysbiosis in AIH patients. In addition, our study also showed that FMT might control hepatitis progression by reversing TFR/TFH cell disorders and restoring the antibiotic-induced IM dysbiosis in EAH mice. These data revealed important information that FMT might be an effective method for the treatment of AIH. However, we recognized the limitations of our study, such as a relative small sample size, variable of water content in individual stools, and the lack of mechanism research of FMT regulating TFR/TFH cell balance in the pathogenesis of AIH. Hence, we are interested in further exploring the aggregate mechanism of FMT regulating TFR/TFH cell balance in the pathogenesis of AIH with a bigger population.

## Data Availability Statement

The datasets presented in this study can be found in online repositories. The names of the repository/repositories and accession number(s) can be found in the article/supplementary material.

## Ethics Statement

The studies involving human participants were reviewed and approved by the Department of Medical Examination Center of the First People’s Hospital of Changzhou. The patients/participants provided their written informed consent to participate in this study. The animal study was reviewed and approved by the Ethical Committee of the First People’s Hospital of Changzhou.

## Author Contributions

ML, ZL, and SJ contributed equally to this work. CJ and ZL designed the experiments and analyzed data. ML wrote the main manuscript text. ML, ZY, and DJ performed the experiments and prepared the figures. WC provided samples. SJ and DF provided conceptual and technical advices. All authors contributed to the article and approved the submitted version.

## Funding

This study was supported by grants from the Changzhou Sci&Tech Program (Grant Nos. CZQM2020011 and CZQM2020079), Natural Science Foundation of Xinjiang Uygur Autonomous Region (2021D01F64), Xinjiang Kirgiz Autonomous Prefecture Medical and Health Technology Project (Grant Nos. 39 and 41), the National Natural Science Foundation of China (Nos. 81700500 and 81700575), the Applied Basic Research Programs of Science, Technology Department of Changzhou City (CJ20160031, CJ20190095 and CJ20210109), the Research Project of Jiangsu Province Commission of Health and Family Planning (No. H201547), the Major Scientific and Technological Project of Changzhou City Commission of Health and Family Planning (No. ZD201612), and the Scientific and Technological Project of Nanjing medical University (No. 2017NJMU042).

## Conflict of Interest

The authors declare that the research was conducted in the absence of any commercial or financial relationships that could be construed as a potential conflict of interest.

## Publisher’s Note

All claims expressed in this article are solely those of the authors and do not necessarily represent those of their affiliated organizations, or those of the publisher, the editors and the reviewers. Any product that may be evaluated in this article, or claim that may be made by its manufacturer, is not guaranteed or endorsed by the publisher.
